# Impact of Maternal Melatonin Suppression on Amount and Functionality of Brown Adipose Tissue (BAT) in the Newborn Sheep

**DOI:** 10.3389/fendo.2014.00232

**Published:** 2015-01-06

**Authors:** Maria Seron-Ferre, Henry Reynolds, Natalia Andrea Mendez, Mauricio Mondaca, Francisco Valenzuela, Renato Ebensperger, Guillermo J. Valenzuela, Emilio A. Herrera, Anibal J. Llanos, Claudia Torres-Farfan

**Affiliations:** ^1^Facultad de Medicina, Laboratorio de Cronobiología, Instituto de Ciencias Biomédicas (ICBM), Universidad de Chile, Santiago, Chile; ^2^Programa de Fisiopatología, Facultad de Medicina, Instituto de Ciencias Biomédicas (ICBM), Universidad de Chile, Santiago, Chile; ^3^Facultad de Medicina, Laboratorio de Cronobiología del Desarrollo, Universidad Austral de Chile, Valdivia, Chile; ^4^Department of Women’s Health, Arrowhead Regional Medical Center, San Bernardino, CA, USA

**Keywords:** development, constant light, circadian, SCN, thermoregulation, newborn

## Abstract

In human and sheep newborns, brown adipose tissue (BAT) accrued during fetal development is used for newborn thermogenesis. Here, we explored the role of maternal melatonin during gestation on the amount and functionality of BAT in the neonate. We studied BAT from six lambs gestated by ewes exposed to constant light from 63% gestation until delivery to suppress melatonin (LL), six lambs gestated by ewes exposed to LL but receiving daily oral melatonin (12 mg at 1700 h, LL + Mel) and another six control lambs gestated by ewes maintained in 12 h light:12 h dark (LD). Lambs were instrumented at 2 days of age. At 4–6 days of age, they were exposed to 24°C (thermal neutrality conditions) for 1 h, 4°C for 1 h, and 24°C for 1 h. Afterward, lambs were euthanized and BAT was dissected for mRNA measurement, histology, and ex vivo experiments. LL newborns had lower central BAT and skin temperature under thermal neutrality and at 4°C, and higher plasma norepinephrine concentration than LD newborns. In response to 4°C, they had a pronounced decrease in skin temperature and did not increase plasma glycerol. BAT weight in LL newborns was about half of that of LD newborns. *Ex vivo*, BAT from LL newborns showed increased basal lipolysis and did not respond to NE. In addition, expression of adipogenic/thermogenic genes (UCP1, ADBR3, PPARγ, PPARα, PGC1α, C/EBPβ, and perilipin) and of the clock genes Bmal1, Clock, and Per2 was increased. Remarkably, the effects observed in LL newborns were absent in LL + Mel newborns. Thus, our results support that maternal melatonin during gestation is important in determining amount and normal functionality of BAT in the neonate.

## Introduction

The unusual profile of melatonin in fetal circulation, provided by the mother during fetal life – and its absence in the early newborn – suggests that maternal melatonin contributes to modulate several functions of key importance in fetal physiology or in preparation for extrauterine life. Along these lines, several studies demonstrate a role of maternally derived melatonin in fetal adrenal function, fetal hippocampus, and fetal heart gene expression, and importantly, its prenatal absence has long-term effects in the offspring (1–5).

A recent observation by our group suggests that maternal melatonin during gestation may also play an important role in brown adipose tissue development and newborn thermoregulation. We found that capuchin monkey newborns from mothers chronically exposed to constant light during pregnancy (treatment that suppress maternal melatonin), had a lower 24-h mean body temperature. This was normalized in newborns whose mothers equally exposed to constant light, received a daily dose of melatonin (6). Since the 24-h mean of the temperature rhythm represents the balance between heat production and heat loss, we speculated that some of these mechanisms were altered by fetal melatonin deprivation.

At birth, the neonate needs to maintain central temperature when facing the transition from the *in utero* environment (over 39°C) to the cooler postnatal environment. Basic mechanisms are turned on at birth to increase heat production and heat conservation. In precocious newborns like sheep, heat production by brown adipose tissue (BAT), accounts for about half of the heat needed by newborns to maintain central temperature, the remaining being produced by muscle thermogenesis (7). BAT mitochondria possess uncoupling protein 1 (UCP1), which uncouples oxidative phosphorylation of fatty acids to produce heat under noradrenergic stimulation (8). BAT is accrued during fetal life in human and sheep newborns (9, 10). The major deposit of BAT in fetal sheep is the perirenal adipose tissue, which is extensively innervated by the sympathetic nervous system and expresses adrenoceptor beta 3 (ADBR3) (9, 11). However, *in vivo*, the response to norepinephrine (NE) is absent during fetal life in BAT. This is attributed to placental or maternal factors, incompletely known (12, 13). In support of maternal melatonin being one of them, we found that fetal perirenal adipose tissue expresses functional melatonin receptors and that melatonin directly inhibits the lipolytic response to NE (14). At birth, when connection with the placenta/mother is severed, exposure to cold stimulates NE release leading to hydrolysis of the triglycerides (TAG), stored in BAT cells, to glycerol and fatty acids (8, 15). Heat production by BAT cells in response to cold, concurrent vasomotor mechanisms for heat distribution, heat conservation, and dissipation (11) and the ability to sustain a circadian temperature rhythm entrained to environmental time cues (6, 16), allow the precocious newborn to keep central temperature in the postnatal environmental conditions.

In the present study, we explored the role of prenatal melatonin on newborn thermoregulation utilizing the lamb as a model. The pregnant sheep and her fetus provide the opportunity to conduct chronic studies in a diurnal species that, like humans, typically produces one or two offspring, similar to the human in weight and several developmental milestones including a prenatal developmental of brown adipose BAT (10). We utilized an integrative approach to study the role of maternal melatonin deprivation and replacement during gestation in the newborn lamb perirenal adipose tissue. We measured thermogenic and lipolytic response (glycerol) on exposure to cold *in vivo*; assessed the perirenal adipose tissue weight and the response to NE in perirenal adipose tissue explants *ex vivo* and determined the perirenal adipose tissue gene expression *in vitro*.

## Materials and Methods

### Animals

All experimental protocols were reviewed and approved by the Committee for Animal Bioethics, Faculty of Medicine, University of Chile (CBA No. 01479) and FONDECYT (Project FONDECYT 1060766). Animal care, maintenance, procedures, and experimentation were performed in accordance with the Guide for the Care and Use of Laboratory Animals published by the US National Institutes of Health (NIH Publication No. 85-23, revised 1996) and adheres to APS’s Guiding Principles in the Care and Use of Animals.

**Figure 1:**
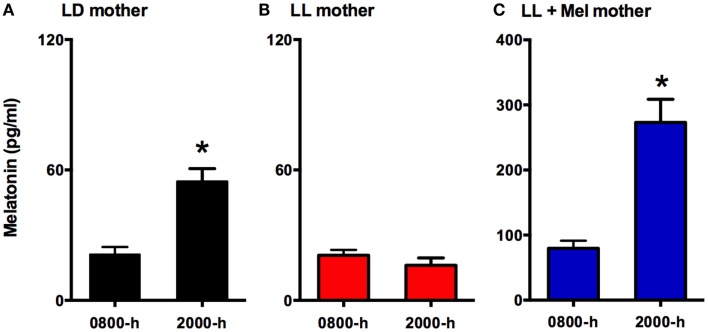
**Means ± SEM plasma melatonin concentration at 0800 and 2000 hrs in pregnant ewes at 90% gestation**. LD ewes (*n* = 5) **(A)** were kept in photoperiod 12:12, lights on at 0700 hrs; LL ewes (*n* = 6) **(B)** were kept under continuous light from 60% gestation and LL + Mel ewes (*n* = 6) **(C)** were kept in continuous light but received an oral dose of 12 mg melatonin at 1700 hrs. *Different to 0800 h, *p* < 0.05 (paired *t*-test).

Eighteen newborns lambs aged 5–6 days were studied. Twelve of these newborns were gestated in ewes maintained in constant light from 63% gestation until delivery (term 147 ± 5 days). Six of these mothers received daily 12 mg melatonin [General Nutrition Corporation (GNC), Pittsburgh, PA, USA] at 1700 h until delivery. Tablets were given orally mixed with grass and the other ewes received only grass. Chronic exposure to constant light suppressed the maternal melatonin rhythm (Figure 1), preventing the increase melatonin concentration seen at 2000 in ewes kept in LD. Daily replacement with 12 mg melatonin given at 1700 h resulted in concentrations at 2000 h about threefold those found at 2000 h in LD mothers. Melatonin concentration decreased reaching values at 0800 similar to those found in control ewes at 2000 h. Another group of six dams were maintained in a 12 h light:12 h dark light cycle (LD, lights on at 0700). Food and water were provided *ad libitum* during gestation and postpartum. All newborns were fed by their mothers and maintained in LD cycle after birth.

Newborns were weighed and clinically examined at birth and daily until the day of the experiment by one of the DVM of the team. There were no effects of the maternal treatments upon weight or clinical conditions at birth and 5 days of age. Birth weight (means ± SD) were: 3.85 ± 0.265 kg in newborns gestated in LD, 3.68 ± 0.33 kg in newborns derived from dams whose melatonin was suppressed by exposure to constant light (LL newborns) and 4.3 ± 0.18 kg in newborns from dams exposed to constant light receiving a daily melatonin replacement (LL + Mel newborns).

### Experimental protocols

#### Thermogenic and endocrine response to cold

At 2 days of age, the lambs were premedicated with atropine (0.04 mg/kg im; Atropina Sulfato; Laboratorio Chile, Santiago, Chile). All surgical procedures were performed under general anesthesia with ketamine, 10 mg kg^−1^ im (Ketostop; Drag Pharma-Invectec, Santiago, Chile) and diazepam 0.1–0.5 mg kg^−1^ im (Laboratorio Biosano, Santiago, Chile) with additional local infiltration of 2% lidocaine (Dimecaina; Laboratorio Beta, Santiago, Chile). Polyvinyl catheters (1.2 mm internal diameter) were placed into the descending aorta and inferior vena cava and a Swan-Ganz catheter (Edwards Swan-Ganz 5 French, Baxter Healthcare Corporation) was placed in the pulmonary artery. A thermistor (MLT-415, Powerlab, ADinstruments, Dunedin, New Zealand) was placed in perirenal adipose tissue. All catheters and the thermistor were exteriorized and kept in a pouch sewn onto the skin. Ampicillin 10 mg kg^−1^ iv (Ampicilina, Laboratorio Best-Pharma), were given every 12 h while the animals were instrumented (17). Once recovered from surgery, newborns returned to their mother. At 4–6 days of age newborns were brought to the laboratory, placed in a sling inside a clear acrylic box and a skin thermistor (MLT-409A) was attached to a previously shaved spot in the rump skin. The box was connected through separated outlets to a heater and to a cooling system. In preliminary experiments, we verified thermoneutral temperature (temperature at which oxygen consumption was lowest) in a separated group of control newborns aged 4–11 days. In our experimental conditions, we found the lowest oxygen consumption occurred between 24.0 and 26.5°C and that newborns appeared comfortable at this temperature range. In the present study, newborns were exposed for 1 h at 24°C (basal period), followed by 1 h at 4°C (cold period) and 1 h at 24°C (recovery period) to assess thermogenic and lipolytic response to cold exposure *in vivo*. During the experiment arterial aortic and pulmonary blood samples were taken every 15 min to measure cardiorespiratory variables to determine arterial pH, PO_2_, PCO_2_, hemoglobin concentration (Hb), and percentage saturation of hemoglobin (SaO_2_) [IL-Synthesis 25 (Instrumentation Laboratories, Lexington, MA, USA); measurements corrected to 39°C]. The oxygen content was calculated from aortic and pulmonary arterial samples. Cardiac output was determined just after the blood sampling by the thermodilution method as the average of three determinations after injection of 3 ml of chilled (0°C) NaCl 0.9% into the pulmonary artery (model COM-2 cardiac output computer, Baxter, Irvine, CA, USA. The neonatal oxygen consumption (VO_2_) was calculated by the Fick’s method:

$$VO_{2}=O_{2}\;\mathrm{content}\;\left(\mathrm{ascending}\;\mathrm{aorta}-\mathrm{pulmonary}\;\mathrm{vein}\right)\times \mathrm{cardiac}\;\mathrm{output}.$$

Systemic arterial pressure was measured continuously using a pressure transducer and recorded by a data acquisition system (Powerlab/8SP System and Chart v4.1.2 Software; ADInstruments, NSW, Australia) connected to a personal computer. Heart rate and mean systemic arterial blood pressure (MAP) were obtained from this record. Central temperature was measured every 15 min (when measuring cardiac output), whilst skin temperature, perirenal fat temperature, and mean arterial blood pressure was measured continuously.

When blood samples were taken for pH and blood gases, an additional amount was obtained to determine plasma glycerol, NE, glucose, thyroid hormones (T3 and T4), and cortisol concentrations.

Experiments started at 1000 h. Upon completion of the previous experimental protocol, newborns were euthanized with an overdose of sodium thiopenthone 100 mg kg^−1^ iv (Tiopental; Laboratorio Biosano, Santiago, Chile). Perirenal adipose tissue and fetal organs were dissected and weighed and the position of the perirenal thermistor was confirmed. Pieces of perirenal adipose tissue were stored in TRIzol at 4°C for molecular biology measurements; others were fixed in paraformaldehyde for histology. In addition, pieces of fresh perirenal adipose tissue were cut in explants to perform the tissue culture experiments reported below.

#### Perirenal adipose tissue lipolytic response to norepinephrine *in vitro*

Perirenal adipose tissue dissected from individual newborns, was cut in small explants (about 25 mg), which were mixed and suspended in culture medium (D-MEM F12, Sigma-Aldrich, St. Louis, MO, USA). Triplicate explants were pre-incubated in culture medium for 6 h at 37°C and aerated with 95% air CO_2_, then explants were incubated in triplicate for 12 h in 2 ml medium alone (basal) or containing 0.01, 0.1, 1, and 10 μM of NE (Levofed 1 mg/ml, Hospira Inc., McPherson, KS, USA). At the end of incubation, the supernatant was collected, explants were weighed and lipolysis was determined measuring the glycerol present in the supernatant fraction. Production of glycerol was calculated as microgram per milligram of tissue and expressed as percentage production by basal explants.

### Assays

Plasma cortisol concentration was measured by RIA (1). Maternal melatonin concentration in plasma was measured melatonin antiserum (AB/S/02, Stockgrand Ltd, Guildford Surrey, UK) and [O-methyl-3H]melatonin (TRK798, Buckinghamshire, UK) as a tracer, following the manufacturer’s recommendations. The inter and intra assay coefficients were <15%. Plasma and supernatant glycerol was measured by the glycerol oxidase method as reported previously (14) using a working reagent prepared by Valtek Diagnostics (Santiago, Chile) containing glycerolkinase, glycerolphosphate oxidase, peroxidase, and ATP. Glucose was measured by the glucose oxidase method with a kit (Glucosa LS, Valtek Diagnostics, Stgo. Chile). T3/T4 was measured by RIA using kits (T3: DSL-3100 ACTIVE; T4: DSL-3200 ACTIVE, DSL Inc., Webster, TX, USA) following the manufacturer’s recommendations. Plasma NE was measured by HPLC in a commercial laboratory (Barnafi Krause Diagnóstica, Santiago, Chile).

Gene expression was measured by qRT-PCR. Primers reported in the literature were used to measure UCP1 (18), adrenergic receptor β3 (ADBR3), PGC1α, PPARγ, PPARα (19), C/EBPβ (20), perilipin (21), and the clock genes Per1 (22), Bmal1, Clock, and Cry2 (23). Per2 primers were designed by us (For: 5′-agc aag tga aag cca gtg agg agt-3′, Rev: 5′-cag cgg cca caa aca tat cca cat-3′). 18SrRNA (24) was used as housekeeping gene. Adipose tissue samples (about 100 mg) were homogenized in TRIzol and the RNA was extracted by the procedure of the SV Total RNA Isolation Kit System (Promega), which includes a DNase treatment. RNA obtained was resuspended in nuclease-free water and the absorbance was measured at 260 and 280 nm. The ratio 260/280 was 1.9–2.05. The RNA was stored at −20°C. Reverse transcription was performed from approximately 0.3 μg of RNA with 100 ng of primers (Random primers) and 200 U M-MLV RT (200 U/μL) in a final volume of 20 μL. Transcription conditions were 65°C for 5 min, 4°C for 5 min, 37°C for 2 min, 24°C for 10 min, 37°C for 50 min, and 70°C for 15 min.

#### 

##### Quantitative real time PCR

Assays were performed in a StepOne thermal cycler from Applied Biosystems, CA, USA. The quantification of samples was performed using a standard curve constructed with serial dilutions of known quantities of PCR products for each gene. These products were prepared by conventional PCR, purified, amplified quantified by densitometry against a 300 bp DNA marker of known concentration and finally stored in aliquots at −20°C. Non-template controls were included in every PCR reaction and a cDNA pool was included to assess inter assay variability. The threshold cycle (Ct) of each sample and the internal control was interpolated in the respective standard curve. The results were expressed as the ratio gene/18SrRNA. Values obtained were normalized calculating the percentage change of each treatment respect to control.

### Data analysis

Data are expressed as means ± SEM. Data were analyzed by ANOVA for repeated measures or ANOVA and the *post hoc* Newman–Keuls or Tukey tests as appropriate. Statistical analyses were performed using GraphPad Prism 4.00 (GraphPad software, Inc.). Differences were considered significant when *P* < 0.05.

## Results

### Basal cardiorespiratory and hormonal data

Maternal exposure to constant light or constant light + melatonin replacement did not affect newborn weight at birth nor at 5 days of age. Newborns appeared clinically healthy. Cardiorespiratory variables, pH, cortisol, T3, and T4 measured under thermal neutrality conditions (24°C), showed similar values in the three groups of newborns (Table 1). These variables changed similarly during exposure to 4°C and again during recovery (re-exposure to 24°C) in the three groups of newborns (data not shown). However, the maternal treatment resulted in differences in other variables between newborns that became evident during thermal neutrality and cold exposure.

**Table 1:** **Means ± SEM of cardiorespiratory variables and plasma hormones in 5 days old lambs during thermoneutrality**.

Group	LD	LL	LL + M
pH	7.44 ± 0.01	7.44 ± 0.01	7.43 ± 0.02
pCO_2_ (mmHg)	37.64 ± 1.05	37.63 ± 0.95	41.4 ± 2.9
pO_2_ (mmHg)	79.67 ± 1.47	80.48 ± 2.27	77.33 ± 1.89
Hb (g/100 ml)	11.03 ± 0.62	10.54 ± 0.69	9.648 ± 0.61
%Sat Hb	90.56 ± 0.71	87.48 ± 0.71	88.70 ± 1.94
VO_2_ (ml/kg/min)	17.44 ± 0.10	18.25 ± 0.92	15.29 ± 1.14
Cortisol (ng/ml)	31.84 ± 2.36	30.8 ± 3.39	33.65 ± 2.8
T3 (ng/ml)	2,48 ± 0.13	2.63 ± 0.14	2.82 ± 0.14
T4 (ng/ml)	63.26 ± 3.05	68.68 ± 5.81	71.07 ± 3.71

*LD, newborns gestated in ewes exposed photoperiod 12:12; LL, newborns gestated in ewes kept in continuous light from 60% gestation to term and LL + Mel, newborns gestated in ewes kept in continuous light but receiving an oral dose of 12 mg melatonin at 1700 h. *N* = 4 per group. VO_2_ = oxygen consumption. Measurements were done at 30, 45, and 60 min of exposure to 24°C*.

### Melatonin deprived newborns were colder than LD newborns under thermal neutrality conditions and responded abnormally to cold

**Figure 2:**
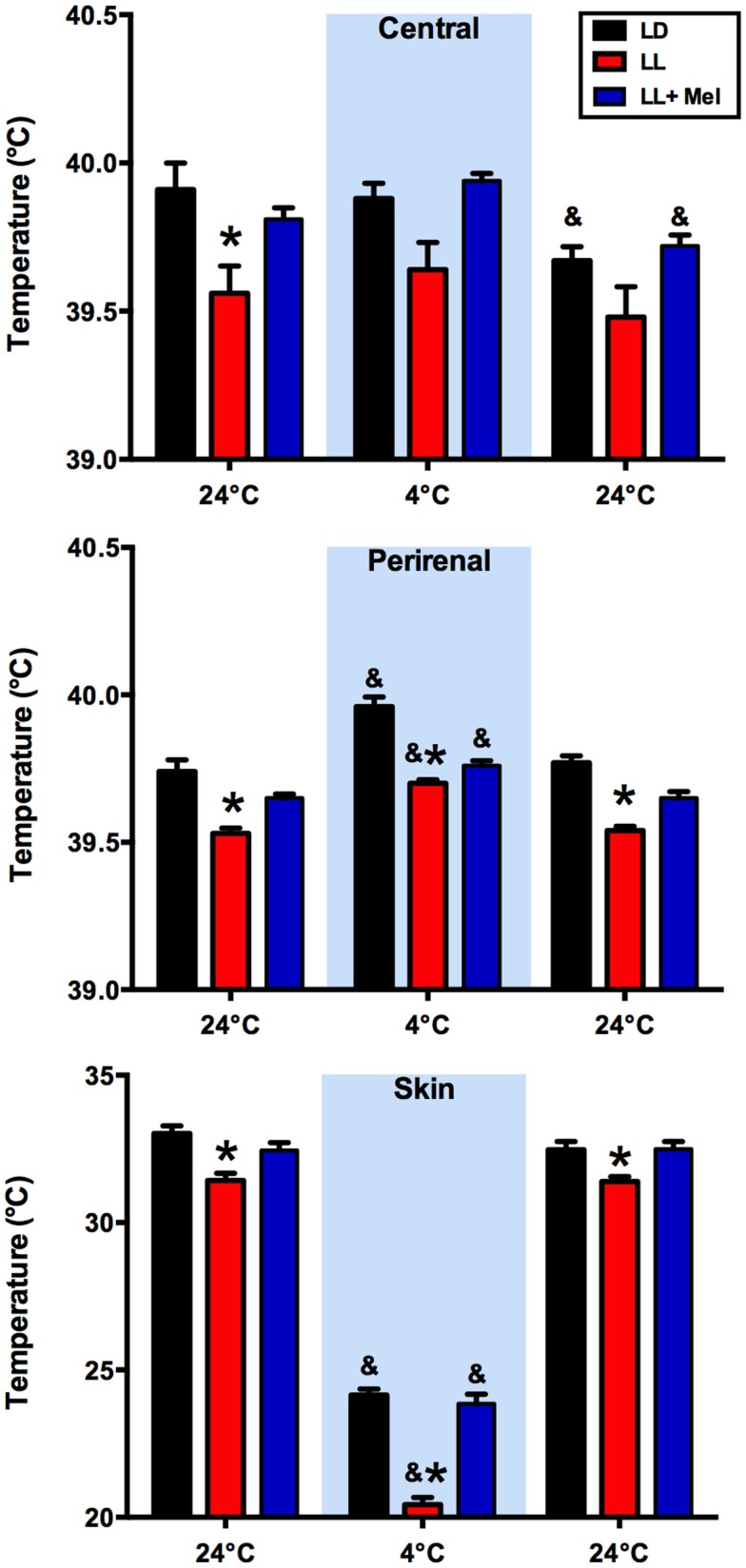
**Response of central, skin, and perirenal temperature to exposure to 4°C in 5 days old lambs**. Data are means ± SEM. LD, newborns gestated in ewes exposed to photoperiod 12:12; LL, newborns gestated in ewes kept in continuous light from 60% gestation to term and LL + Mel, newborns gestated in ewes kept in continuous light but receiving an oral dose of 12 mg melatonin at 1700 h. LD and LL *n* = 6. LL + Mel *n* = 4. *P < 0.05 vs. LD; ^&^*P* < 0.05 vs. first values at 24°C. ANOVA and Tukey’s test.

We measured central, skin, and perirenal fat temperature under thermal neutrality conditions (24°C), and under exposure to 4°C to stimulate facultative thermogenesis. These three temperatures were lower in LL newborns than in LD and LL + Mel newborns under thermal neutrality conditions, suggesting that melatonin deprivation during fetal life affected some mechanisms of thermoregulation in the newborns (Figure 2). The three groups of newborns maintained central body temperature during the hour of exposure to 4°C at the level observed at 24°C. However, the difference in skin temperature between LL and LD newborns increased over twofold during cold exposure. Perirenal temperature increased during exposure to 4°C in the three groups of newborns, returning to basal values at the end of the cold exposure period. However, a difference was present in the duration of the response to cold. Whereas perirenal temperature was maintained between 30 and 60 min of cold exposure in LD newborns, this temperature decreased during the second 30 min of exposure to 4°C, in LL newborns although temperatures values were still higher than during thermal neutrality. LL + Mel newborns showed an increase in perirenal temperature that lasted only 30 min (data not shown).

**Figure 3:**
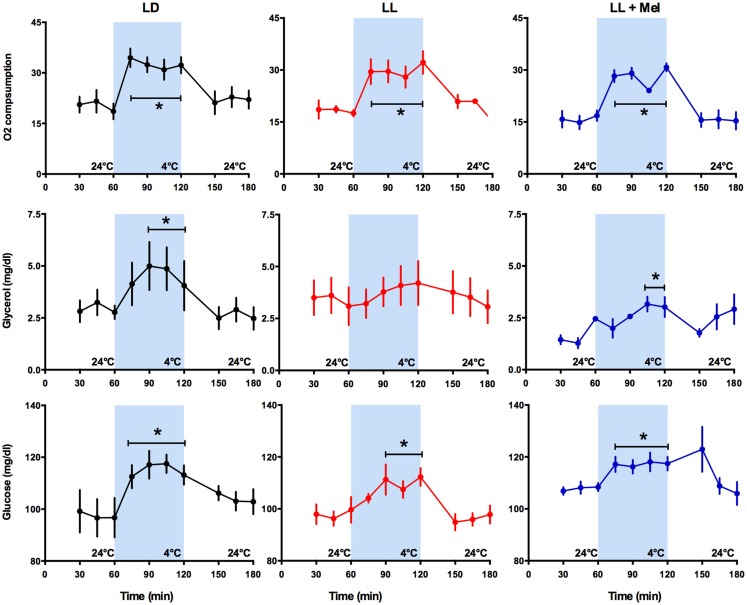
**Response of O_2_ consumption and plasma glycerol and glucose to exposure to 4°C in 5 days old lambs**. Data are means ± SEM, LD, newborns gestated in ewes exposed photoperiod 12:12; LL, newborns gestated in ewes kept in continuous light from 60% gestation to term and LL + Mel, newborns gestated in ewes kept in continuous light but receiving an oral dose of 12 mg melatonin at 1700 h. *N* = 6 per group. *P < 0.05 vs. values at 24°C. ANOVA for repeated measures and Tukey’s test.

The newborn lamb maintains central temperature when exposed to cold by producing heat and reducing heat loss. Overall heat production is measured by oxygen consumption (VO_2_). Exposure for 1 h to 4°C induced a marked increase in VO_2_ in the three groups of newborns, reaching a similar maximal value and returning to basal values at the cessation of cold exposure (Figure 3).

Known responses to cold in the newborn lamb at the systemic level are increases in plasma NE concentration, in plasma glycerol due to brown adipose tissue lipolysis and in plasma glucose concentration (15). As shown in Figure 3, LL newborns did not respond to cold with the expected increase in plasma glycerol concentration, although such response was present in LD and LL + Mel newborns (Figure 3). Lambs neonates from mothers exposed to constant light plus melatonin had a lesser but significant increase in plasma glycerol in response to cold. Glucose concentrations at 24°C were slightly lower in LL newborns than in LD newborns whereas LL + Mel newborns showed a slightly higher glucose concentration (104.2 ± 2.5, 97.9 ± 2.2, and 107.8 ± 0.98 mg/dl, LD, LL, and LL + Mel, respectively, *P* < 0.05, ANOVA and Tukey’s test). The response of glucose to exposure to 4°C was similar in the three groups of newborns (Figure 3).

**Figure 4:**
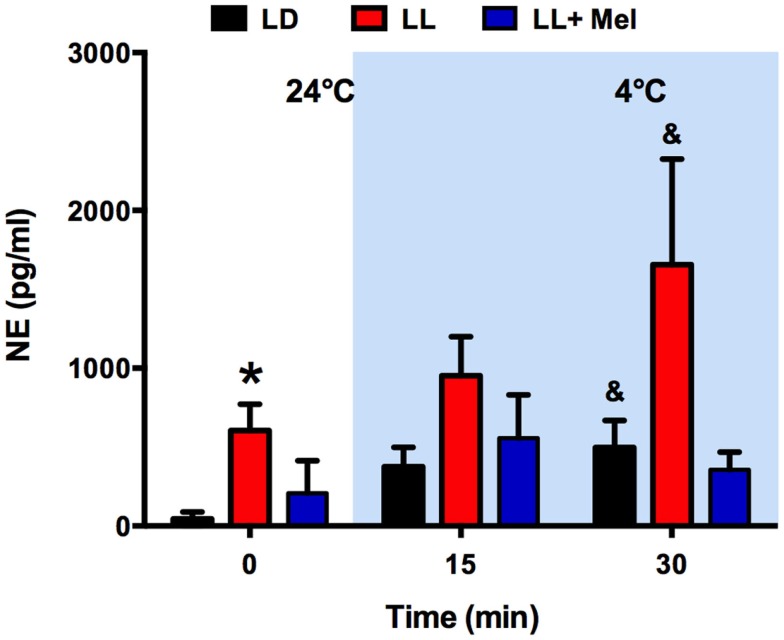
**Response of plasma norepinephrine (NE) concentration to exposure to 4°C in 5 days old lambs**. Data are means ± SEM. LD, newborns gestated in ewes exposed to photoperiod 12:12; LL, newborns gestated in ewes kept in continuous light from 60% gestation to term and LL + Mel, newborns gestated in ewes kept in continuous light but receiving an oral dose of 12 mg melatonin at 1700 h. *N* = 4 per group. Samples were taken at the end of 1 h exposure to 24°C, and at 15 and 30 min of exposure to 4°C. *P < 0.05 vs. LD; ^&^*P* < 0.05 vs. values at 24°C. ANOVA and Tukey’s test.

As shown in Figure 4, the absence of glycerol response to cold in LL newborns occurred in the presence of a marked increase in plasma NE. Plasma NE concentration, measured at 24°C, immediately before initiation of exposure to 4°C, was higher in LL newborns than in LD newborns. Exposure to 4°C induced an increase in plasma NE in the LD and LL newborns, which became significant at 30 min of exposure, whereas no significant increase was detected in LL + Mel newborns.

### Melatonin deprived newborns had less perirenal adipose tissue, which differs from perirenal fat of LD and LL + Mel newborns in response to NE and in gene expression

**Figure 5:**
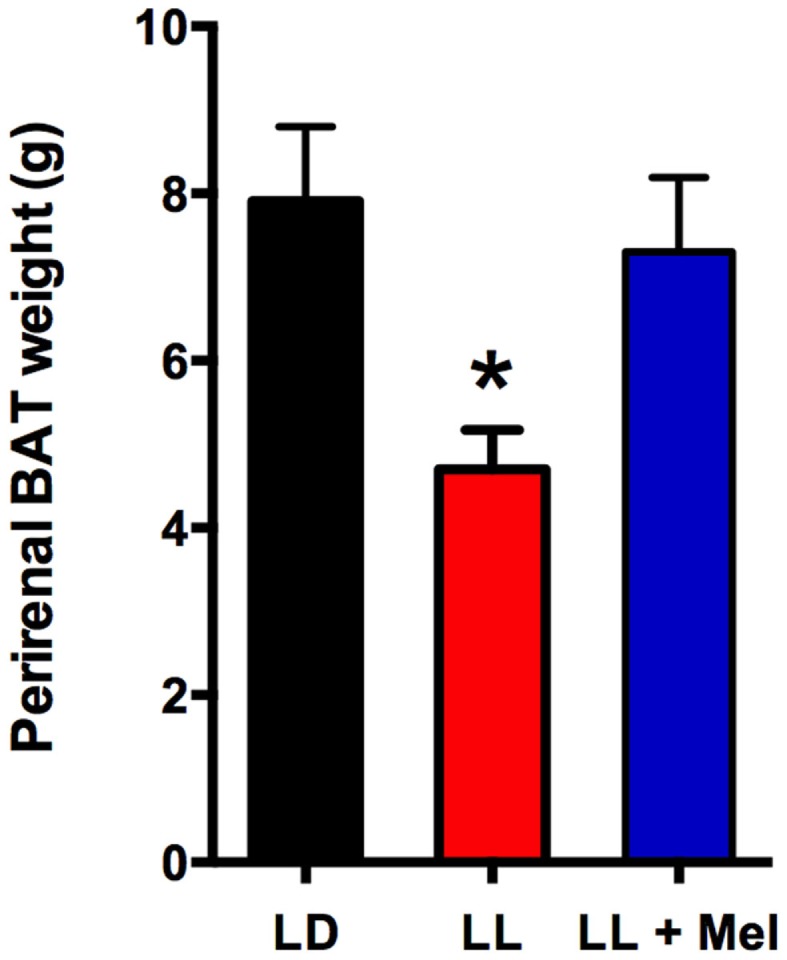
**Perirenal adipose tissue weight in 5 days old lambs**. Data are means ± SEM, LD, newborns gestated in ewes exposed photoperiod 12:12; LL, newborns gestated in ewes kept in continuous light from 60% gestation to term and LL + Mel, newborns gestated in ewes kept in continuous light but receiving an oral dose of 12 mg melatonin at 1700 h. *N* = 6 per group. *P < 0.05 vs. LD; ANOVA and Tukey’s test.

Upon completion of the *in vivo* experiments, perirenal adipose tissue was dissected and weighed. A striking finding was that perirenal adipose tissue weight was lower in LL newborns compared to LD and LL + Mel newborns (Figure 5).

**Figure 6:**
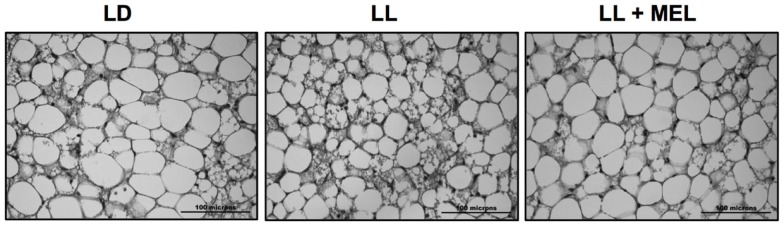
**Representative microphotographs of perirrenal adipose tissue from a 5 days old LD lamb**. LD, newborns gestated in ewes exposed to photoperiod 12:12; LL, newborns gestated in ewes kept in continuous light from 60% gestation to term and LL + Mel, newborns gestated in ewes kept in continuous light but receiving an oral dose of 12 mg melatonin at 1700 h.

Perirenal fat in the 5 days old newborn is composed of patches of multilocular (brown) adipocytes intermingled with unilocular (white) adipocytes (9). These two types of cells were present in perirenal fat in the three experimental conditions tested in the present study (Figure 6) thus our measurements reflect both types of cells.

**Figure 7:**
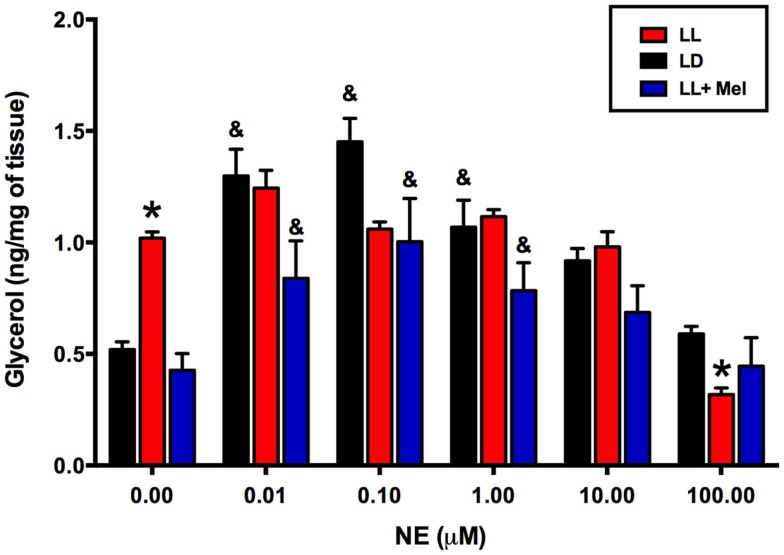
**Glycerol response to increasing doses of NE of perirenal adipose tissue explants from 5 days old lambs**. Data are means ± SEM. LD, newborns gestated in ewes exposed to photoperiod 12:12; LL, newborns gestated in ewes kept in continuous light from 60% gestation to term and LL + Mel, newborns gestated in ewes kept in continuous light but receiving an oral dose of 12 mg melatonin at 1700 h. *N* = 6 per group. *P < 0.05 vs. LD; ^&^*P* < 0.05 vs. 0 NE. ANOVA and Tukey’s test.

Using adipose tissue explants, we detected differences between the groups in the *in vitro* response of perirenal adipose tissue to NE. As shown in Figure 7, unstimulated glycerol production was higher in the perirenal adipose tissue explants obtained from LL newborns than in those of LD and LL + Mel newborns. Moreover, adipose tissue from LL newborns did not show the increase in glycerol production in response to increasing doses of NE observed in perirenal adipose tissue explants from LD and LL + Mel newborns.

**Figure 8:**
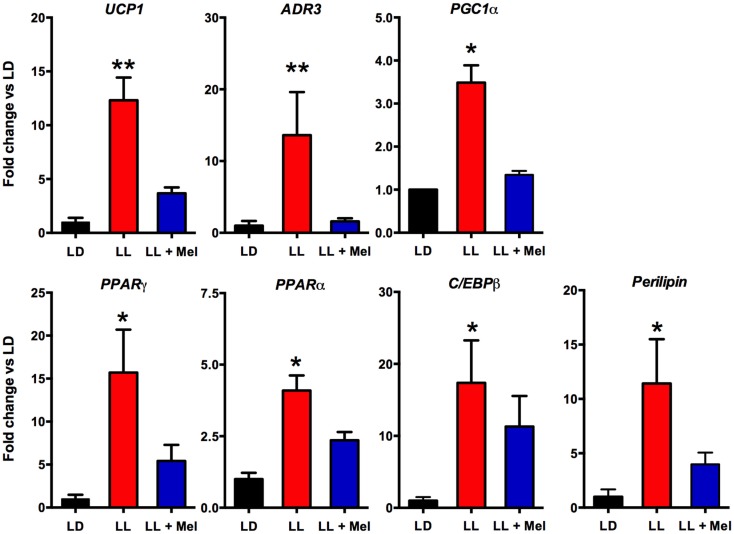
**Expression of adipose tissue genes (means ± SE fold changes vs. LD newborns) in 5 days old newborns**. LD, newborns gestated in ewes exposed to photoperiod 12:12; LL, newborns gestated in ewes kept in continuous light from 60% gestation to term and LL + Mel, newborns gestated in ewes kept in continuous light but receiving an oral dose of 12 mg melatonin at 1700 h. LD and LL *n* = 6. LL + Mel *n* = 4. *P < 0.05 vs. LD; **P < 0.05 vs. LD; and LL + Mel, ANOVA, and Tukey’s test.

**Figure 9:**
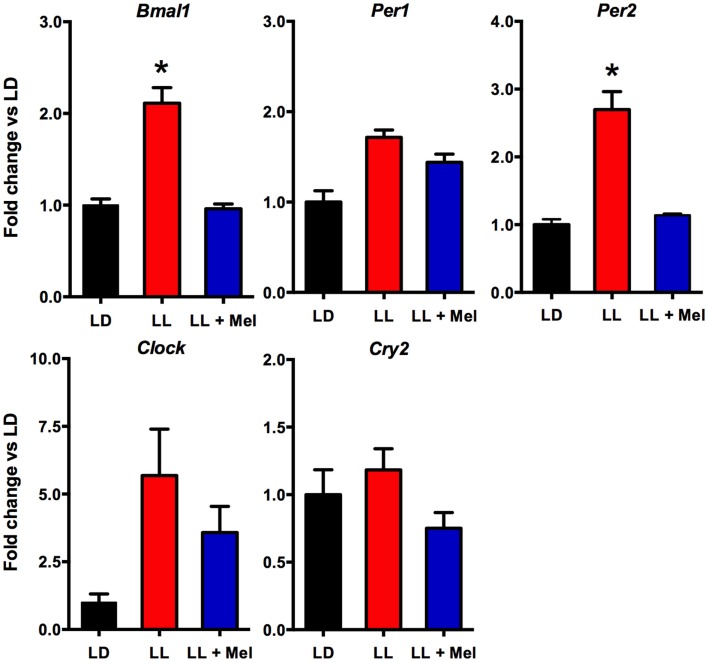
**Expression of clock genes (means ± SE fold changes vs. LD newborns) in 5 days old newborns**. LD, newborns gestated in ewes exposed photoperiod 12:12; LL, newborns gestated in ewes kept in continuous light from 60% gestation to term; and LL + Mel, newborns gestated in ewes kept in continuous light but receiving an oral dose of 12 mg melatonin at 1700 h. LD and LL *n* = 6. LL + Mel *n* = 4. *P < 0.05 vs. LD, **P < 0.05 vs. LD, and LL + Mel, ANOVA, and Tukey’s test.

Additional differences were detected in gene expression (Figures 8 and 9). We measured genes related to thermogenesis (UCP1, ADBR3, and PGC1α) and adipogenesis (PPARγ, PPARα, and C/EBPβ) (9) and of perilipin associated with packing of lipid droplets and lipolysis (25). In addition, we measured the expression of clock genes Bmal1, Per1, Per2, Clock, and Cry2 known to be expressed in brown and white adipose tissue (26–28). As shown in Figures 8 and 9, all genes but Per1, Clock, and Cry2 showed a higher expression in perirenal fat from LL newborns. The increases in UCP1, ADBR3, Bmal1, and Per2 were reversed in perirenal adipose tissue of LL + Mel newborns.

## Discussion

In previous studies, we have investigated physiological roles served by the daily passage of melatonin from the mother to the fetus in several species. Our general hypothesis has been that that melatonin may influence fetal functions critical for neonatal adaptation to extrauterine life. The present experiments investigated the role of maternal melatonin during gestation on preparing the perirenal adipose tissue of newborns lambs for postnatal thermogenesis. We studied newborn lambs chronically deprived of maternal melatonin during their last third of fetal life by exposing pregnant ewes to constant light. We found that at 5 days of age, these newborns were colder than control newborns, had increased plasma NE levels and did not increase lipolysis in response to cold exposure. Moreover, the amount of perirenal adipose tissue was decreased to half, showed an increased basal lipolysis and did not respond to NE *in vitro*, confirming the *in vivo* findings. In addition, the expression of genes related to both white and brown adipocytes, was enhanced suggesting an overstimulated perirenal adipose tissue. The effects of maternal melatonin deprivation were minimized in newborns whose mothers received melatonin during gestation while being exposed to constant light. Overall, these findings substantiate a role of prenatal maternal melatonin in the preparation of postnatal perirenal adipose function.

The increased levels of plasma NE and the decrease in perirenal fat weight in LL newborns were unexpected. In the lamb and other newborns, plasma NE concentration increases markedly at birth and remains elevated during the first 10 days of life (29). In newborns and adults plasma NE, represents overall spillover of NE, resulting from the balance between secretion and reuptake of NE from peripheral sympathetic terminals in most newborn organs and clearance from plasma (30, 31). Thus, the elevated NE levels in LL newborns may represent the continuation of increased NE levels already present during fetal life. We cannot attribute this solely to melatonin as in our experiments plasma NE in newborns of LL + Mel mothers show intermediate values to those of LL and LD newborns. Spontaneous and high altitude chronic hypoxia and growth retardation increase NE levels in the fetus and newborn (32, 33). These factors were not present in our experiments judging from the lack of effects of the treatments in body weight and cardiorespiratory variables found at 5 days of age. Although our experiments were not designed to study the sympathetic nervous system, they suggest that melatonin exerted a restraining role for NE during fetal life, either at central or peripheral levels. Melatonin receptors are present in several areas of the fetal brain including areas that may be involved in sympathetic nervous system control in human, sheep, rat, and in newborn pigs (34–37). In adult hamsters, MEL 1a mRNA was co-localized with SNS outflow neurons that innervate white fat (38). Several studies in the adult human and rats show acute effects inhibitory of melatonin in the modulation of the sympathetic system ranging from decreases in plasma NE levels and blood pressure (39, 40) to lowering the response to orthostatic stress and decreasing muscle and skin sympathetic nerve response to several stimuli (41–43). Since the lamb perirenal adipose tissue is heavily innervated by the SNS, increased pre and post natal sympathetic stimuli may impact adipose tissue amount, promoting lipolysis rather than lipogenesis. Indeed, overstimulation with a β adrenergic agonist, ritodrine but not with NE, increased lipolysis as indicated by increased in fetal plasma fatty acids and decreased fetal perirenal adipose tissue weight (44). *In vitro*, melatonin inhibits NE stimulated lipolysis (14). As we will discuss below, basal lipolysis of perirenal adipose tissue explants from LL newborns is augmented. We suggest that in LL newborns, melatonin deprivation resulted in an unrestrained increased sympathetic tone favoring lipolysis and reducing perirenal adipose tissue weight.

We assessed overall effects of the treatments over temperature regulation by measuring central, skin, and perirenal fat temperature under thermal neutrality conditions (24°C), situation at which facultative thermogenesis is minimal in the lamb and under exposure to 4°C to stimulate facultative thermogenesis. In these two environmental conditions, we found lower temperatures in LL newborns than in LD and LL + Mel newborns, suggesting that melatonin deprivation during fetal life affected newborn thermoregulation. In the newborn lamb, Williams and coworkers demonstrated that during cold exposure the contribution of perirenal adipose tissue heat production, accounts for about 40% of the increase in oxygen consumption used to defend central temperature, the remaining heat being produced by muscle contraction (shivering). In our experiments, oxygen consumption was similar in the three groups of newborns studied at 24°C and increased similarly at 4°C, thus total heat production was similar in each group. Perirenal adipose tissue temperature increased in LL newborn exposed to cold, compared with the temperature of this tissue at 24°C. However, total contribution to heat production of this tissue would be lower, considering its decrease in size, thus other mechanisms possibly shivering (not quantified in our experiments) accounted for the increased oxygen consumption. We assessed further the contribution of perirenal adipose tissue on heat production by measuring cold induced lipolysis *in vivo* and NE induced lipolysis *in vitro*. LL newborns responded to a cold challenge with an increase in NE and plasma glucose but not with an increase in plasma glycerol, suggesting limited induction of lipolysis *in vivo* by NE. The *in vitro* studies, performed after the cold exposure experiment, confirm these findings and demonstrate almost a doubling of basal glycerol production suggesting that adipose tissue was already maximally stimulated in absence of added NE. This increased basal glycerol production per milligram tissue may have little impact on plasma concentration given the reduction in adipose tissue weight. Exposure to cold, in addition to stimulate heat production, triggers mechanisms that decrease heat loss. One of them is skin vasoconstriction that reduces skin temperature. In our experiments, skin temperature was already about 1.6°C lower in LL than in LD newborns while at 24°C suggesting an important vasoconstriction. Of note, the difference in skin temperature between LL and LD newborns increased over twofold during cold exposure, indicating that skin vasoconstriction plays an important role in maintenance of central temperature in LL newborns. Whether the increased in NE levels discussed above are involved it is not known. Overall, these results suggest that in LL newborns, the reduced amount of perirenal adipose tissue, although competent to produce heat makes a minor contribution to temperature defense, the task being shifted to other mechanisms.

We next investigated possibly changes on gene expression on the adipose tissue that could relate to the above findings. We found that the expression of UCP1, ADBR3, PPARγ, C/EBPβ, and perilipin increased about 10-fold, and PGC1α, PPARα, and the clock genes Per2 and Bmal1 increased about 4–6-fold in perirenal adipose tissue from LL newborns. PPARγ is central to brown and white adipose function and directly controls the expression of genes involved in lipid transport, lipid metabolism, insulin signaling, and adipokine production (45). In addition, it controls perilipin expression (46). Of note, over expression of perilipin in mice white adipose tissue results in increased basal lipolysis (25). Per2 protein is a component of the negative branch of circadian clocks and has been shown to interact with nuclear receptor proteins in the liver, providing a link between metabolic activity and circadian function (47). Such interaction has been shown for Per2 and PPARg in white adipose tissue (27) and Per2 and PPARa in brown (26); functional results of these studies were an increase in UCP1 expression, among other genes. Changes in UCP1, ADBR3, BMAL1, and Per2 gene expression were completely reversed, and PPARγ and perilipin partially reversed in LL + Mel newborns’ perirenal tissue. The increases in gene expression in adipose tissue from LL newborns may relate to the increased NE stimulation discussed previously as shown for UCP1 and PGC1α in the perirenal adipose tissue of 5 days old lambs (24), and for Bmal1, UCP1, and PGC1α in adult mice brown fat (28). Recent studies, also in adult mice brown fat, showed that cold exposure increases Per2 and Adbr3 expression (26). On the other hand, direct effects of melatonin on clock gene expression have been demonstrated in the adrenal of several species (48–50) and in striatral neurons *in vitro* (51). Given that functional binding sites for melatonin are present in fetal perirenal adipose tissue and that melatonin directly inhibits NE stimulated lipolysis, we suggest that the combined effects of increased NE stimulation plus prenatal melatonin deprivation may explain increased gene expression on perirenal adipose tissue.

Previous work from our laboratory and others showed the involvement of melatonin during gestation in perinatal adrenal function in primates and rats (1, 2), in rat hippocampal expression of NMDA receptors, and fetal cardiac genomics (3, 4), with long-term consequences for the offspring spatial memory (4) and energy metabolism (5). In the present study, we extend maternal melatonin role to the adipose tissue and suggests a wider role including the sympathetic nervous system. Altogether, these evidences highlight the importance of maternal melatonin during pregnancy for the newborn.

Regarding the potential relevance of the present findings to human health, it must be kept in mind that exposure to light at night (which effectively suppresses melatonin), most probably is a perinatal environmental risk factor imposed by a modern 24/7 society. In this context, our data demonstrate negative effects of maternal melatonin suppression during pregnancy in the newborn lamb, a species very relevant for studies on perinatal physiology. Whether this might carry on into adulthood as abnormal physiological traits, is a possibility that needs to be seriously considered, since there is a body of evidence suggesting that a deleterious maternal environment increases the risk of developing diseases like diabetes, hypertension, obesity, and metabolic syndrome that appear in adult life (52).

## Conflict of Interest Statement

The authors declare that the research was conducted in the absence of any commercial or financial relationships that could be construed as a potential conflict of interest.
